# DNA methylation analysis improves the prognostication of acute myeloid leukemia

**DOI:** 10.1002/jha2.187

**Published:** 2021-03-13

**Authors:** Hanie Samimi, Isha Mehta, Thomas Roderick Docking, Aamir Zainulabadeen, Aly Karsan, Habil Zare

**Affiliations:** ^1^ Department of Computer Science Texas State University San Marcos Texas USA; ^2^ Department of Cell Systems & Anatomy The University of Texas Health Science Center San Antonio Texas USA; ^3^ Canada's Michael Smith Genome Sciences Centre British Columbia Cancer Research Centre Vancouver British Columbia Canada; ^4^ Department of Computer Science Princeton University Princeton New Jersey USA; ^5^ Glenn Biggs Institute for Alzheimer's and Neurodegenerative Diseases University of Texas Health Sciences Center San Antonio Texas USA

## Abstract

Integration of orthogonal data could provide new opportunities to pinpoint the underlying molecular mechanisms of hematologic disorders. Using a novel gene network approach, we integrated DNA methylation data from The Cancer Genome Atlas (*n *= 194 cases) with the corresponding gene expression profile. Our integrated gene network analysis classified AML patients into low‐, intermediate‐, and high‐risk groups. The identified high‐risk group had significantly shorter overall survival compared to the low‐risk group (*p*‐value ≤$10^{-11}$). Specifically, our approach identified a particular subgroup of nine high‐risk AML cases that died within 2 years after diagnosis. These high‐risk cases otherwise would be incorrectly classified as intermediate‐risk solely based on cytogenetics, mutation profiles, and common molecular characteristics of AML. We confirmed the prognostic value of our integrative gene network approach using two independent datasets, as well as through comparison with European LeukemiaNet and LSC17 criteria. Our approach could be useful in the prognostication of a subset of borderline AML cases. These cases would not be classified into appropriate risk groups by other approaches that use gene expression, but not DNA methylation data. Our findings highlight the significance of epigenomic data, and they indicate integrating DNA methylation data with gene coexpression networks can have a synergistic effect.

## 1 INTRODUCTION

Acute myeloid leukemia (AML), which is the most common acute leukemia among adults [1], has several subtypes that have various prognoses [2] depending on age, cytogenetic abnormalities [3], specific mutations [4], and other unknown risk factors (Note S1). Timely diagnostics of AML is critical for determining the best approach to clinical management [5].

**FIGURE 1:**
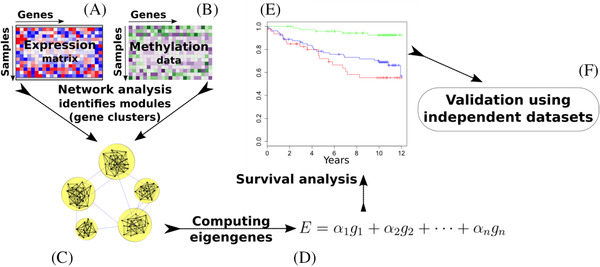
**Schematic view of the methodology**. The inputs include **(a)** a gene expression profile and **(b)** a DNA methylation profile. **(c)** We build an integrative network that models the association between individual gene pairs based on both expression and methylation data using Equation 1 (Supplementary Note 3). **(d)** For each module, we compute an eigengene as a weighted average of the expression of all genes in that module. **(e)** We use the eigengenes as robust biological signatures (biomarkers) to perform survival analysis. **(f)** The results are validated using independent cohorts of AML

We previously showed that coexpression gene network analysis is useful to identify subtle but consistent signatures of AML that otherwise would be difficult to pinpoint using conventional differential expression analysis [6]. In the current study, we tested the hypothesis that integrating DNA methylation data into the model can be useful in detecting prognostic signatures, because more robust modules would be identified (Figure 1) [7, 8]. To the best of our knowledge, this is the first study on systematic integrating of DNA methylation and transcriptome data in a single gene network. Using this approach, we developed a prognostic test based on data from The Cancer Genome Atlas (TCGA) consortium [9] (*n *= 194 cases), and we validated our findings using two independent AML cohorts.

## 2 METHODS

### 2.1 Description of training and validation datasets

We used TCGA data to train our model (i.e., build our gene network and select the prognostic modules). We downloaded gene expression (*n *= 179) and DNA methylation (*n *= 194) data from the TCGA AML publication web page [9]. To validate our findings, we used two independent datasets: (1) the German Acute Myeloid Leukemia Cooperative Group (AMLCG) 1999 Cohort (*n *= 562, microarray) [10 ^–^ 13] and (2) the Beat AML RNA‐Seq dataset (*n *= 405) [14].

The AMLCG cohort: We downloaded the gene expression profiles of 562 AML samples from Gene Expression Omnibus (series number GSE37642) [15]. We obtained the corresponding clinical data, including overall survival, life status, and risk category based on the European LeukemiaNet (ELN‐2010) [16] from the authors.

**Highlights**

1. Incorporating DNA methylation into gene network analyses results in more robust and informative gene modules.
2. Using an integrative gene network approach, we identify high‐risk AML cases that otherwise would be classified as intermediate‐risk.

The Beat cohort: We obtained the reads per kilobase of transcript per million mapped [17] (RPKM) values (i.e., the gene expression profile) and the survival data of the Beat AML cohort from the supplementary tables of the corresponding publication [14]. We transformed the RPKM values using natural logarithm [18]. In our survival analysis, we included only those 405 cases whose reported time from diagnosis to death is non‐zero.

### 2.2 Preprocessing data

Our approach can benefit from all DNA methylation data in constructing the network even if gene expression data are missing for some of the corresponding cases. We excluded genes and loci (i.e., DNA methylation probes) that did not pass the quality control criteria, showed too little variation, or had too weak correlation with overall patient survival. We used only the 24,649 loci that showed an absolute Pearson correlation of 0.2 or higher with overall survival. These loci correspond to 9377 genes, and the majority of these genes (95%) were associated with four loci at most (Figure S1). We then computed the effective DNA methylation level for each gene (Note S2).

### 2.3 Gene network analysis

After data preprocessing, 12,535 genes were determined to have some relevance with overall survival based on either expression, or DNA methylation levels. To identify groups of genes that are associated with each other (i.e., gene modules), we built a network in which each node corresponds to one of these 12,535 genes (Figure 1). We computed the similarity between each gene pair based on both expression and DNA methylation levels. Specifically, for each gene pair, we computed (1) the Pearson correlation between their expression levels and (2) the Pearson correlation between their DNA methylation levels. We combined the resulting correlation values to derive the similarity (i.e., association) between each pair of genes (Note S3).

In our integrative gene network, each node represents a gene, and the edge between two genes (i.e., their connection) is weighted based on their similarity. We identified groups of similar genes (i.e., modules) using a hierarchical clustering approach [19] (Note S4). For each gene module, we then computed an eigengene based on a principal component analysis [6, 20] (Note S5) and used these eigengenes to delineate the modules that are associated with overall survival of AML (Note S6).

### 2.4 Comparison with other prognostication approaches

Gerstung et al approach provides continuous risk estimates [21]. To systematically compare this approach with our prognostic test, we discretized Gerstung et al risk estimates (i.e., we considered the first quartile low‐risk, the second and third quartiles intermediate‐risk, and the fourth quartile high‐risk). We also compared our approach with European LeukemiaNet genetic risk stratification and the LSC17 score, which is based on a leukemic stem cell (LSC) signature [22] (Note S7).

### 2.5 Data availability

All data analyzed during this study are included in the Supplementary Information files, which can be used to reproduce our results.

## 3 RESULTS

### 3.1 Integrative network analysis

Using our novel integrated network analysis (Figure 1, Methods), we identified 78 modules (clusters) of genes (Figure S4). The genes in each module are highly associated with each other with respect to their DNA methylation and gene expression patterns (Table S1). For each module, we computed an eigengene, which is a weighted average of the expression levels of the genes in that module (Note S5 and Table S2). Module 56 had the eigengene that showed the strongest correlation with overall survival (Pearson correlation *r *= 0.3), while Module 51 had the most anticorrelated eigengene (*r *= 0.4). In this correlation analysis, each module was considered individually. To determine whether a subset of modules together would be more useful for the prognostication of AML, we performed a survival analysis using Cox regression.

### 3.2 Survival analysis

**FIGURE 2:**
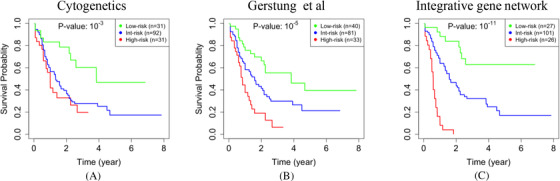
**The Kaplan‐Meier** [25] **(KM) curves for all cases in the TCGA‐AML dataset**. The log‐rank *p*‐values indicate that differences between the low‐risk group (green) and the high‐risk group (red) are statistically significant. Compared to cytogenetics criteria (**a**), the approach developed by Gerstung et al (**b**) led to a lower *p*‐value (i.e., 10*^−^* ^5^ *< *10*^−^* ^3^). Our gene network analysis, which includes gene expression and epigenome data but not mutation data, resulted in a *p*‐value of 10*^−^* ^11^ (**c**). In particular, all 26 cases that we identified as high‐risk (i.e., 17% of the entire cohort) died within 2 years after diagnosis. Validation on two independent cohorts confirmed that the prognostic value of our analysis is not specific to the TCGA‐AML dataset, which we used to train our model

Among the participants in the TCGA cohort, the prevailing cytogenetic criteria classified 31 cases as low‐risk, 92 as intermediate‐risk, and 31 as high‐risk cases (Figure 2a) [9]. To determine whether our integrative gene network analysis can improve prognostication, we used the 78 inferred eigengenes as covariates (i.e., potential prognostic features) in a penalized Cox regression analysis [23, 24]. We found that the most associated subset of three modules with overall survival included Modules 46, 51, and 55 (Note S6). Some biological pathways are overrepresented in these three modules [26].

Module 46 has 19 genes (Table S1) with a significant overlap with the following pathways: (1) the immune system based on Reactome [27], (2) the signaling events mediated by class II histone deacetylases (HDAC) based on Pathway Interaction Database [28], and (3) the signaling by nerve growth factor [29] based on Reactome (hypergeometric test *p*‐values = 0.001). The overlaps between these pathways and Module 46 include the following genes previously reported relevant to AML: *CAMK4* [30, 31], *IL6ST* [32], *SOCS2* [33, 34], *TUBB2A* [35 ^–^ 37], and *BCL2L11* [38]. While the expression of these genes anticorrelates with survival time, the overlaps also include *DUSP3* [39, 40], which correlates with survival time.

Module 51 has 15 genes with a significant overlap with the post‐translational protein modification (*p*‐values = 0.001) and metabolism of proteins (*p*‐values = 0.005) pathways. The overlaps include *PLAUR* (*CD87*) [41 ^–^ 44] with positive, and *PMM1* with negative, correlations with survival time, respectively.

Module 55 has 14 genes with a significant overlap with the following Reactome pathways: (1) apoptosis [45], (2) membrane trafficking [46], and (3) cellular responses to stress [47] (*p*‐values = 0.01). The overlaps include *YWHAH* [48], *GPX1* [49], and *UBC*. The expression of these genes was negatively correlated with survival time.

To evaluate the significance of these selected modules in predicting overall survival, we fitted an *accelerated failure time* model to the selected eigengenes [50, 51]. Based on this model, we predicted the expected survival time of each patient and then classified them into low‐, intermediate‐, and high‐risk groups (Table S3). There was a significant difference between the survival times of the low‐and high‐risk groups that we identified (*p*‐value ≤ 10*^−^* ^11^, Figure 2c). When we stratified the patients into two age groups, the difference between the identified low‐ and high‐risk groups was still significant in the group of patients who were diagnosed with AML after age 60 (*n *= 74, *p*‐value ≤ 0.004), and also in the rest of relatively younger patients (*n *= 92, *p*‐value ≤ 10*^−^* ^6^, Figure S5). As expected, we identified more high‐risk (*n *= 19) and fewer low‐risk (*n *= 5) patients in the older group compared to the relatively younger group (*n *= 7 and *n *= 22, respectively). There were 15 males and 11 females in the high‐risk group, which did not indicate a significant difference between the two genders (hypergeometric test *p*‐value *≥* 0.2).

### 3.3 Enhancement of current prognostication approaches

Because it is challenging to decide on the best treatment for the intermediate‐risk group, we investigated whether combining our analysis with other classification approaches for AML would lead to narrowing down the prognostication of purportedly intermediate‐risk patients. Acute promyelocytic leukemia (APL) is a subtype of AML with a distinct cytogenetic signature, which allows clinicians to quickly and accurately diagnose it using fluorescent in situ hybridization and polymerase chain reaction [52]. Therefore, we excluded the 21 APL patients in the TCGA datasets in the following assessment. The group of 79 non‐APL patients that Gerstung et al classified as intermediate‐risk (i.e., the second and third quartiles based on the predicted survival probability) included a mix of actual high‐ and low‐risk cases. In this group, 47 cases (60%) died of AML, while 14 cases (18%) were relatively low‐risk cases. These 14 low‐risk cases were followed for at least 2 years after diagnosis, and they were found to be alive at the last time of contact. It is thus critical to further assess the clinical risk for this subset of cases.

**FIGURE 3:**
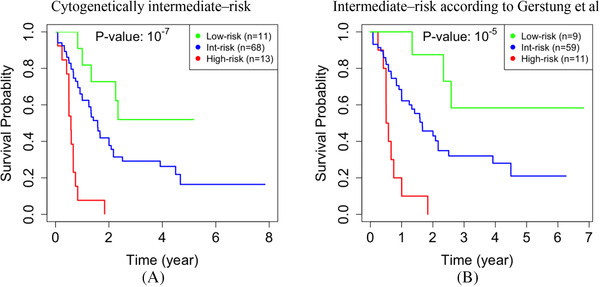
**Restratification of purportedly intermediate‐risk AML cases in the TCGA dataset**. There are 92 cytogenetically intermediate‐risk cases (the blue KM curve in Figure 2a). Using our integrative gene network approach, we further classified these 92 cases into 11 low‐, 66 intermediate‐, and 13 high‐risk cases (**a**). The approach by Gerstung et al would classify 81 cases as intermediate‐risk, with survival probabilities higher than the first quartile but lower than the fourth quartile (the blue KM curve in Figure 2b). After excluding two APL cases, we further classified the remaining 79 non‐APL cases into nine low‐risk, 59 intermediate‐risk, and 11 high‐risk cases (**b**). In each plot, the *p*‐value corresponds to log‐rank tests with a null hypothesis that the predicted high‐risk cases have the same expected survival time as other cases in that plot

We investigated the utility of our approach in the prognostication of the 79 non‐APL cases that would be classified as intermediate‐risk by Gerstung et al (Figure 3b). Our approach identified a subset of 11 high‐risk cases (14%) who survived significantly shorter than other cases in this group (*p*‐value ≤ 10*^−^* ^5^). All of these 11 cases died of AML within 2 years after diagnosis, although the majority of them (i.e., six cases, 55%) had a normal karyotype, two cases had the del(7q) abnormality, and one case had the t(9;11)(p22;q23) translocation. Two cases had complex cytogenetics with three or more distinct abnormalities (Table S3).

### 3.4 Validation using independent datasets

To show that the prognostication performance of the three identified modules is not specific to the TCGA dataset, we validated our findings using the German AMLCG 1999 and Beat cohorts.

**FIGURE 4:**
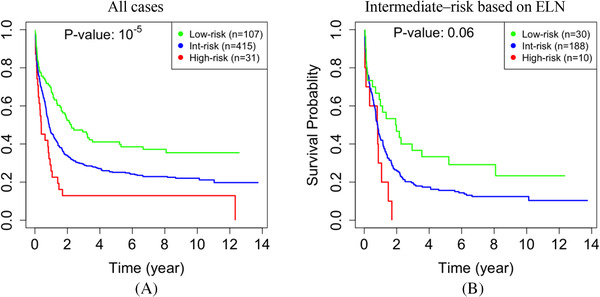
**The KM curves for the AMLCG 1999 cohort**. Cases that we identified as low‐ (green) and high‐risk (red) have significantly different survival times (**a**). While 228 cases had an intermediate‐I or intermediate‐II ELN risk score, our approach determined that 10 of these cases were high‐risk, all of whom died within 2 years after diagnosis (**b**)

**The German AMLCG 1999 cohort**. We identified 107 (19%) low‐, 415 (75%) intermediate‐, and 31 (6%) high‐risk cases in the AMLCG cohort (Table S3). There was a significant difference between the survival time of the low‐ and high‐risk groups that we identified (*p*‐value ≤ 10*^−^* ^5^, Figure 4a). More interestingly, all 10 cases with an intermediate ELN risk score that we identified as high‐risk died within 2 years after diagnosis. This suggests that our approach can identify a subgroup of truly high‐risk cases that would not be considered as such using the current clinical criteria. To further quantify and support this assertion, we compared this group of 10 cases with the other 218 patients in the AMLCG 1999 cohort with an intermediate‐I or intermediate‐II ELN‐2010 risk score. After adjustment for multiple hypothesis testing, there was no significant association among the variables included in the ELN‐2017 genetic risk classifier [53], nor other common clinical variables including white blood cell counts, hemoglobin, platelets, lactate dehydrogenase, and Eastern Cooperative Oncology Group score [54] (*p*‐value *≥* 0.05).

**The Beat cohort**. Of the 405 cases with survival data in the BEAT cohort, 156 (39%) were not classified as favorable or adverse by ELN2017. Using the eigengenes that were identified based on TCGA data, we classified these 156 cases into 62 (40%) low‐, 86 (55%) intermediate‐, and eight (5%) high‐risk cases (Table S3). Interestingly, none of the eight cases that we identified as high‐risk were known to be alive long after diagnosis. That is, five cases died within 1 year after diagnosis, while the other three cases left the study within 16 months (i.e., 458, 302, and 16 days, respectively) after diagnosis. A hypergeometric test showed that if five cases were randomly selected from the pool of 156 cases, it would have been unlikely that all of selected cases die within 1 year after diagnosis (*p*‐value *≤* 0.002).

### 3.5 The significance of DNA methylation in gene network analysis

**FIGURE 5:**
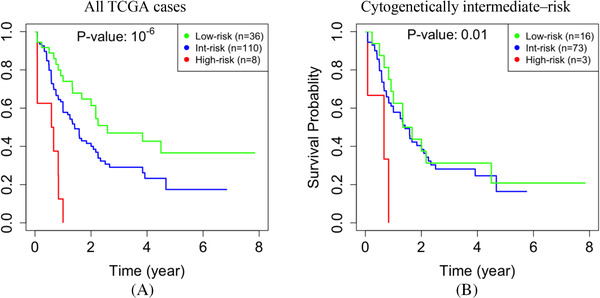
**Network analysis without epigenomic data**. When DNA methylation data were not used in constructing the gene network, the ability to predict survival decreases in the entire TCGA dataset (*p*‐value = 10*^−^* ^6^ vs. 10*^−^* ^11^ in Figure 2c) **(a)**, and in the subset of 92 cytogenetically intermediate‐risk cases (10*^−^* ^2^ vs. 10*^−^* ^7^ in Figure 3a) **(b)**

To assess the significance of epigenomics in our study, we repeated our analysis using gene expression but without DNA methylation data. Without using DNA methylation data, our analysis identified fewer modules, which were generally larger. Specifically, we identified 36 modules, and their sizes had a mean, median, and standard deviation of 339, 38, and 917, respectively. The *p*‐value for separation between the low‐ and high‐risk cases was less significant (10*^−^* ^6^ in Figure 5a vs. 10*^−^* ^11^ in Figure 2c). We also compared the performance of prognostication for the subset of 92 cytogenetically intermediate‐risk cases. The performance decreased when epigenomic data were excluded from the analysis. Specifically, the *p*‐value of 10*^−^* ^2^ in Figure 5b, which is equivalent to a chi‐square statistic of 28, is less significant than the *p*‐value of 10*^−^* ^7^ in Figure 3a, which is equivalent to a chi‐square statistic of 7. In particular, without DNA methylation data, we could identify only eight high‐risk cases, three of which were cytogenetically classified as intermediate‐risk. Collectively, the above results indicate that integrating DNA methylation data into the network is essential for identifying gene modules that are relevant to prognosis.

## 4 DISCUSSION

Almost half of AML cases cannot be confidently classified into low‐ or high‐risk groups using current prognostic criteria. Transcriptome and epigenome data have been used to prognosticate AML (Note S1). Our study showed that integrating these data in a single model can have a synergistic effect and improve prognostication (Figure 5). While both gene expression and DNA methylation data from TCGA cohort were used to identify gene modules, we used only gene expression data from two independent cohorts to validate our findings because DNA methylation data were not available in the later (Note S5). Interestingly, our approach is useful in prognostication of AML in the validation cohorts, although DNA methylation data were not available for these cases. Outperforming LSC17 score, which was developed based on transcriptome data, suggests that integration of epigenome data in the training phase results in more informative gene modules. This supremacy is expected because DNA methylation provides us with additional robust biological information to train the model.

Validation using the AMLCG and Beat AML cohorts showed that the prognostication performance of the three identified modules is not specific to the TCGA dataset. That is, for all studied cohorts, our approach allows for the identification of a particular group of high‐risk cases that otherwise would be inaccurately considered intermediate‐risk based on the current prognostic factors. In particular, when our risk assessment is compared to the approach presented by Ng et al, which is based on LSC [22], our assessment reveals that these high‐risk cases are almost evenly distributed between the low‐and high‐risk groups based on their LSC17 score. This suggests that the modules we identified represent a signature that is distinct from the LSC17 score (Note S7).

Clinical utility. When combined with other integrative schemes, our approach can further improve risk assessment especially to identify some of the high‐risk cases that otherwise would be classified as intermediate‐risk. For instance, if a case is categorized in the gray intermediate‐risk group by other approaches but our assessment suggests that case is high‐risk, then the actual risk for the case is most likely high. Specifically, when combined with the Gerstung et al approach, we could identify 11 (6%) more high‐risk cases in the TCGA dataset (Figure 3b). When combined with ELN‐2010, we could identify 10 (2%) more high‐risk cases in the AMLCG dataset (Figure 4b). Interestingly, these cases exhibited a poor prognosis, with a survival rate of less than 2 years. Our results show that using DNA methylation data in building the gene network has synergistic benefits. In follow‐up studies, integrating other multiomics data into the network could perhaps improve prognostication further.

In conclusion, using an integrative network approach to group thousands of genes into modules, we showed that incorporating DNA methylation into gene network analyses results in more robust and informative gene modules because each resulting module is a group of coexpressed and co‐methylated genes. Some of these modules define survival signatures, which can be combined to develop a prognostic test (Figure 2c).

## CONFLICT OF INTEREST

The authors have declared that no competing interests exist.

## AUTHOR CONTRIBUTIONS

Habil Zare and Aly Karsan conceived the experiments. Hanie Samimi, Habil Zare, Aamir Zainulabadeen, and Thomas Roderick Docking conducted the experiments. Habil Zare and Aly Karsan analyzed the results. Habil Zare, Hanie Samimi, Thomas Roderick Docking, Isha Mehta, and Aly Karsan wrote the manuscript. All authors reviewed the manuscript.

## Supporting information

Supporting Information

Supporting Information
