# Supplementary material for: DNA methylation analysis improves the prognostication of acute myeloid leukemia
Source: EJHaem. 2021 Mar 13;2(2):211–8. doi: 10.1002/jha2.187 (PMC8294109; doi:10.1002/jha2.187)
Supplement: Supplementary file 1 — Supporting Information [file JHA2-2-211-s001.pdf]

DNA methylation analysis improves the prognostication of acute myeloid leukemia

Hanie Samimi[1], Isha Mehta[2], Thomas Roderick Docking[3], Aamir Zainulabadeen[4], Aly Karsan[3,+], and Habil Zare[2,5,+,*]

[1]Department of Computer Science, Texas State University, San Marcos, Texas, 78666, USA
[2]Department of Cell Systems & Anatomy, The University of Texas Health Science Center, San Antonio, Texas, 78229, USA
[3]Canada's Michael Smith Genome Sciences Centre, British Columbia Cancer Research Centre, Vancouver, British Columbia, V5Z 1L3, Canada
[4]Department of Computer Science, Princeton University, Princeton, New Jersey, 08540, USA
[5]Glenn Biggs Institute for Alzheimer's & Neurodegenerative Diseases, San Antonio, Texas, 78229, USA
[+]These senior authors contributed equally to this work.
[*]Corresponding author. Email: zare@uthscsa.edu. Mailing address: Department of Cell Systems & Anatomy, 7703 Floyd Curl Drive, San Antonio, TX 78229, USA. Phone:(210) 567-6797.

List of Supplementary Figures

S1 The distribution of the number of loci per gene. 5
S2 Distance of the selected loci to the closest transcription start site (TSS). 6
S3 Choosing λ based on the best p–value 8
S4 The distribution of module sizes. 10
S5 The KM curves for old and young groups. 13
S6 Performance of LSC17 on intermediate–risk cases. 15

List of Supplementary Notes and Tables

Supplementary Note S1: Current AML prognostication methods 2
Supplementary Note S2: Gene expression and DNA methylation preprocessing . . . 3
Supplementary Note S3: Gene network construction 7
Supplementary Note S4: Identifying gene modules 9
Supplementary Table S1: Module assignments 11
Supplementary Note S5: Computing eigengenes 11
Supplementary Table S2: Eigengenes 11
Supplementary Note S6: Details of survival analysis 11
Supplementary Table S3: Risk assessment 12
Supplementary Note S7: Comparison with LSC17 score 14

Supplementary Note S1: Current AML prognostication methods. Acute Myeloid Leukemia (AML) is a cancer of the myeloid blood cells in which bone marrow produces abnormal blood cells. It is a disease of the elderly and the most common acute leukemia in adults[1]. Timely prognostication of AML is challenging[2,3] due to complex prognostic factors including age, cytogenetic abnormalities[4,5], specific mutations[6–9], and other unknown risk factors. While 35 to 40% of adult patients younger than 60 years of age can be cured using the current standard treatments, the rate of success is limited to in 5 to 15% in older patients[10,11].

Most established prognostication schemes, e.g., the 2016 World Health Organization (WHO) classification of myeloid neoplasms and acute leukemia[12], are based on cytogenetics and specific mutations. Nevertheless, several groups have developed prognostic tests based on gene expression signatures[13–16,16–20,20–40]. Recently, Ng et al analyzed leukemia stem cells (LSC) obtained from 78 AML patients, and proposed the LSC17 score for prognostication of AML[38]. Specifically, they sorted bone marrow samples into CD34+CD38- LSCs and CD34+CD38+ non-LSC fractions[29,41], and identified 104 genes as differentially expressed in these fractions. Using the least absolute shrinkage and selection operator (LASSO)[42,43], they derived a prognostic signature as the weighted sum of expression of an optimal subset of 17 genes. A higher LSC17 score is associated with poorer outcomes in adult[44] and pediatric[45] AML. LSC17 score correlates with percentage of bone marrow blast cells at diagnosis, and also with higher incidence of *FLT3-ITD* mutation and adverse cytogenetic features[46].

Gerstung et al recently reanalyzed 111 cancer genes, and integrated cytogenetic profiles, fusion genes, point mutations, gene–gene interactions, demographic features, clinical risk factors, and treatment received across 1,540 AML cases. Based on these data, they inferred an overall survival probability distribution for each individual case. To compare cytogenetic risk categories with Gerstung et al approach, the cases in the first and fourth quartiles of the predicted overall survival probability distribution were considered as low– and high–risk, respectively. The rest of the cases were considered intermediate–risk. They showed that their integrative approach provides considerably more informative and accurate statements than the current standards in clinical practice[6,47]. In particular, validation using data from independent patients in the TCGA cohort revealed that Gerstung et al approach is superior to the prognostication solely based on cytogenetics (Figure 2b).

Although the critical role of DNA methylation in tissue differentiation and homeostasis has been known for decades[48–50], epigenomic data have only recently been used to classify and prognosticate AML[51–68]. Approaches for integration of DNA methylation with gene expression data include: 1) selecting genes for epigenomic analysis based on gene expression profiling[64], 2) overlapping between differentially methylated and differentially expressed genes[61,62], 3) clustering cases based on DNA methylation and then differential expression analysis of clusters[58], or vice versa[59], 4) using differentially expressed and differentially methylated genes to cluster AML cases[69,70], and 5) characterizing novel AML subtypes that were originally defined based on mutations[56].

Almost half of AML cases cannot be confidently classified into low– or high–risk groups using current prognostic criteria. For example, in the TCGA dataset, 92 cases (51%) would be considered as intermediate–risk based on cytogenetics. However, the survival outcome of these cases is variable, and the actual risk in this group is in fact a mix of low– and high–risk

(Figure 3a). Even when mutations in specific genes and other clinical data were added to the cytogenetic data using the approach of Gerstung et al , 81 TCGA cases (45%) could not be accurately prognosticated (Figure 3b).

Supplementary Note S2: Gene expression and DNA methylation preprocessing. We performed the following steps for quality control and computing gene expression and DNA methylation levels per gene.

Gene expression data analysis

We downloaded reads per kilobase of transcript per million mapped[71] (RPKM) values of 179 AML cases in the The Cancer Genome Atlas (TCGA) dataset from the corresponding publication web page[68]. We excluded the genes with too little variation (i.e., a standard variation less than 10^{-8}). We standardized RPKM values of each gene by subtracting the average RPKM value across all samples, and then dividing the difference by the standard deviation of the RPKM values of that particular gene. We computed the Pearson correlation between the gene expression levels and the survival time of the 133 patients who died of AML. Consistent with the approach taken by other scholars in applying gene network analysis[72–74], we kept the top third ($n = 6,637$) of genes that were most correlated with the survival time in our study.

Prepossessing of DNA methylation data

In the TCGA dataset[68], DNA methylation levels were measured in 485,577 genomic loci (i.e., CpG sites) in 194 AML samples using the Infinium HumanMethylation450 BeadChip array[75]. We excluded the 92,943 loci that had too many missing values, or did not pass the quality control criteria (RnBeads Version 1.10.8[76]). For each of the remaining 392,634 loci, we computed the Pearson correlation[77] between the level of DNA methylation and the survival time of the 133 patients who died of AML. We identified the 24,649 loci that had an absolute Pearson correlation of 0.2 or higher with the survival time. These loci correspond to 9,377 genes (Supplementary Figure S1).

Computing effective DNA methylation levels per gene

We used the following approach to compute the effective DNA methylation level for each gene in the network model. When the number of loci corresponding to a gene was five or less, we used principal component analysis[78] (PCA) to compute the weighted average of the beta value of those loci, and considered the first principal component as the effective DNA methylation level for that gene. When the number of loci corresponding to a gene was more than five, we selected a subset of highly co–methylated loci.

Selecting a subset of highly co–methylated loci When the number of loci corresponding to a gene was more than five, we identified a subset of loci that had correlated DNA methylation levels. That is, we excluded the loci that had little correlation with other loci corresponding to the same gene in the following way. We constructed a graph in which the nodes were the loci corresponding to a gene. The edge between a pair of loci was weighted according to the absolute value of the Pearson correlation between the corresponding beta values. Using the `cluster_fast_greedy` function from the igraph package (Version 1.1.2)[79], we identified the

dense subgraphs (communities). We selected the subgraph with the maximum average weight, and applied PCA on the nodes of that subgraph to compute the level of DNA methylation for the corresponding gene. One third of the selected loci were closer than 1,000 base pairs (bps) to the TSS of a transcript (Supplementary Figure S2).

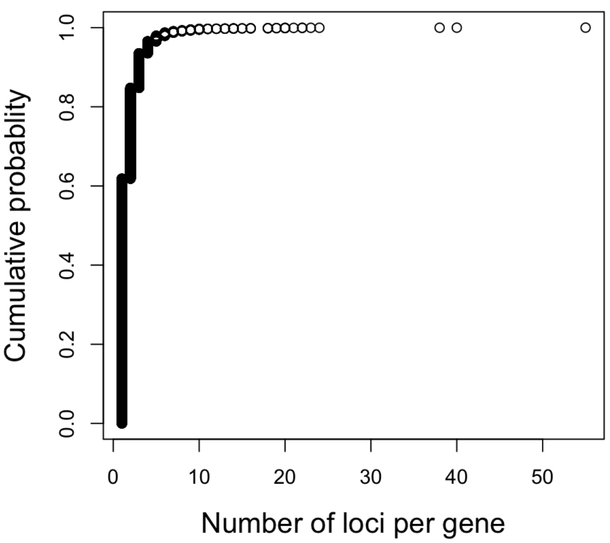

a

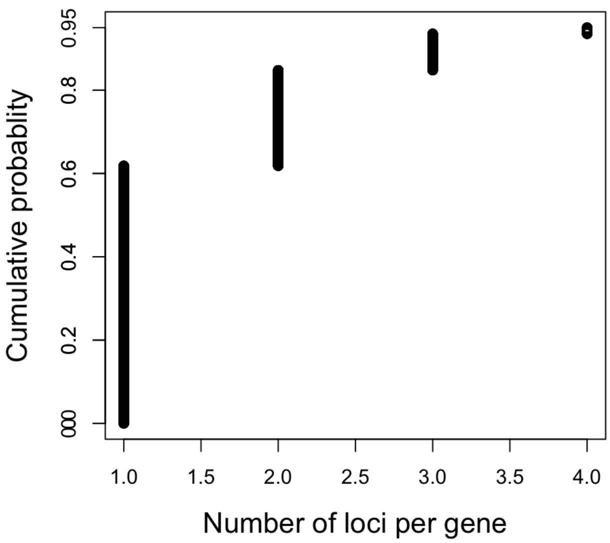

b

Supplementary Figure S1. The distribution of the number of loci per gene.
The cumulative probability distribution of the number of loci per gene, considering only the loci where the DNA methylation levels correlate with the survival time of AML cases in the TCGA dataset. While 6,087 loci do not correspond to any annotated gene, the correspondence between 6,686 loci and annotated genes is one–to–one. **(a)** For the rest of the genes, the number of loci corresponding to each gene varies, and **(b)** it is less than five loci per gene for 95% of genes.

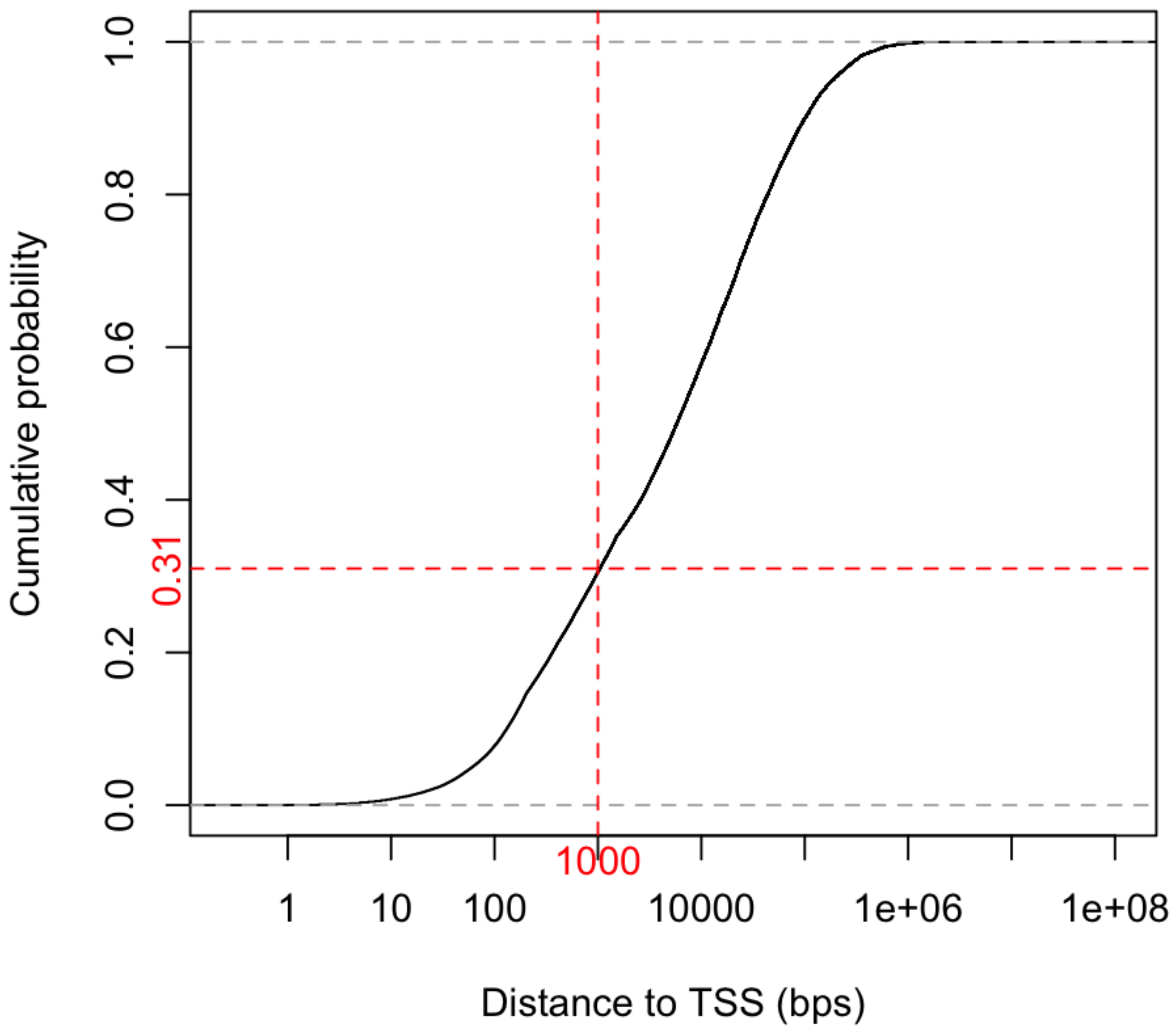

Supplementary Figure S2. Distance of the selected loci to the closest TSS. The x axis shows the distance of the selected loci to the closest TSS measured as the number of base pairs. The y axis is the cumulative probability function. For any particular cutoff distance on the x axis, this curve shows on the y axis, the fraction of the selected loci in this study that are closer than the cutoff distance to a TSS. For example, the red dashed lines indicate that 31% of the selected loci are within 1,000 base pairs of a TSS.

Supplementary Note S3: Gene network construction. The DNA methylation levels of 9,377 genes, and the gene expression levels of 6,637 genes, correlate with survival time. The union of these two sets includes 12,535 genes, on which we performed integrative network analysis. Each of the 12,535 nodes in the network represents a gene. For each gene pair, we computed the Pearson correlation between their expression levels, and also between their DNA methylation levels.

Based on the following formula, we assigned a weight to the edge of the network that connects genes g_i and g_j together (Figure 1c):

$$\mathscr{W}(g_i,g_j) = (1-\lambda)\left|\mathrm{cor}_E(g_i,g_j)\right| + \lambda\left|\mathrm{cor}_M(g_i,g_j)\right|, \tag{1}$$

where $\mathscr{W}(g_i,g_j)$ represents the *integrated* similarity (association) between genes g_i and g_j, $\left|\mathrm{cor}_E(g_i,g_j)\right|$ denotes the absolute value of the correlation between level of expression of two genes, and $\left|\mathrm{cor}_M(g_i,g_j)\right|$ is the absolute value of the correlation between their respective levels of DNA methylation. In this formula, $0 \le \lambda \le 1$ is a hyperparameter that controls the relative effect of each data type. That is, with $\lambda = 0$, the model basically ignores DNA methylation data, and it will be identical to a conventional coexpression network (Figure 5). With a larger λ, the model gives a higher weight to the DNA methylation data. In particular, gene expression levels are ignored if $\lambda = 1$. In this study, we chose $\lambda = 0.6$, which led to the best p–value for high– vs. low–risk stratification in the TCGA dataset (Supplementary Figure S3).

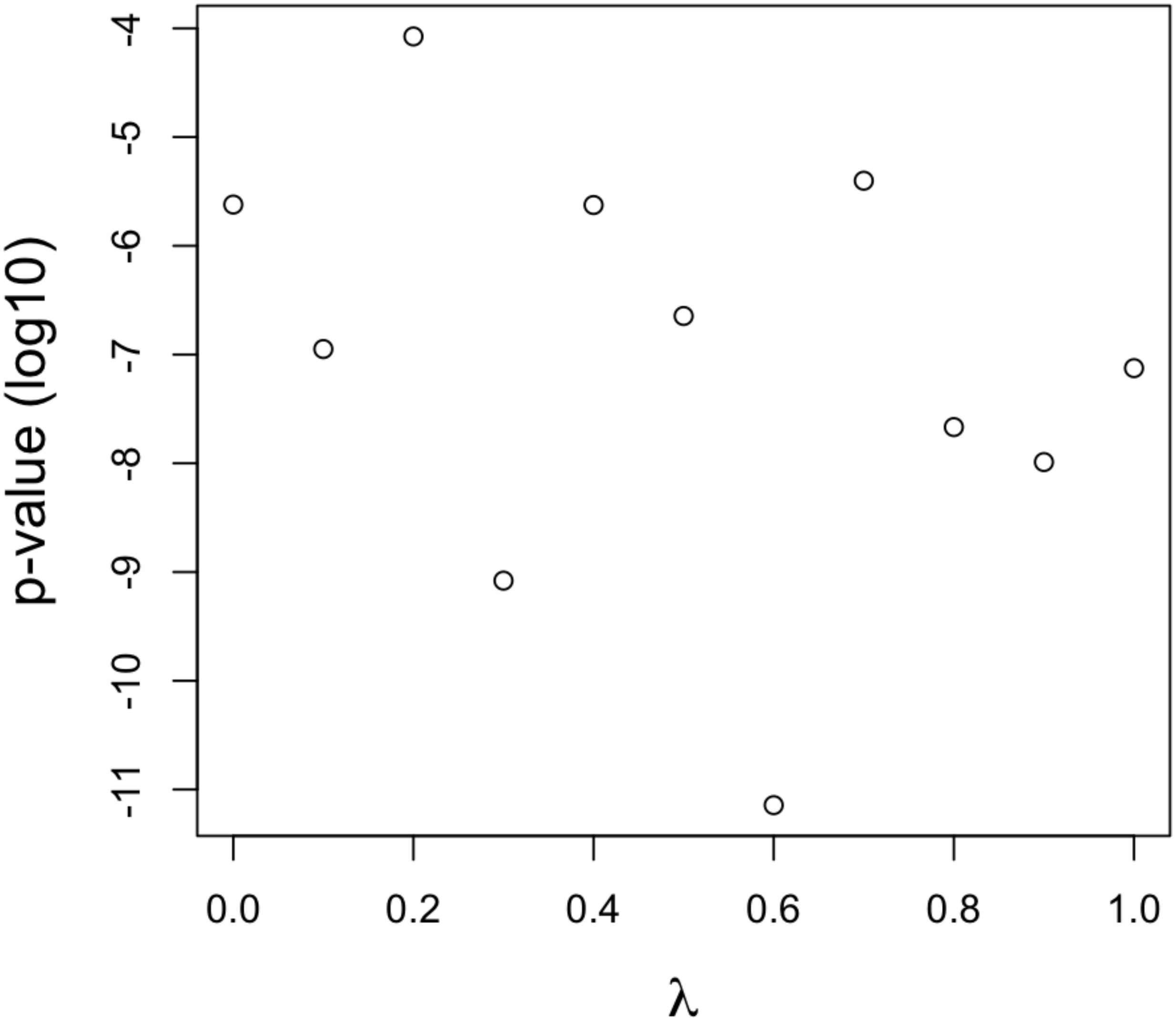

Supplementary Figure S3. Choosing λ based on the best *p*–value . We repeated the integrative gene network analysis in TCGA dataset using Equation 1 with multiple λ values (x–axis), which led to different *p*–values for high- vs. low-risk stratification (y–axis). We chose $\lambda = 0.6$ because this value led to the best *p*–value .

Supplementary Note S4: Identifying gene modules. We included 12,535 genes in the network analysis based on correlation with survival time in the AML data from TCGA[68]. Each node in the network represents a gene. The edge (connection) between a pair of genes is weighted in order to integrate gene expression and DNA methylation data into a single network. Specifically, a higher correlation or anticorrelation between the gene expression levels resulted in a higher weight on the corresponding edge. This weight also increases when the effective DNA methylation levels of the two genes correlate or anticorrelate (Equation 1).

We used the R[80] package WGCNA[81] (Version 1.63) to identify gene modules (clusters) in the integrative network based on a hierarchical clustering approach. Specifically, we used Equation 1 to define the similarity between genes based on the DNA methylation and gene expression levels in the TCGA-AML dataset. Using the `pickSoftThreshold.fromSimilarity` function with the default parameters and a `RsquaredCut` value of 0.75, the power (β) parameter was inferred to be 8[82]. We used the `blockwiseModules()` function to identify gene modules. For better results, we set the parameter `maxBlockSize=12,535` so that the process was performed in only one block. This prevented errors that could have occurred when merging the results from smaller blocks. We set `TOMType="unsigned"` and `minModuleSize=5`, and we used the default values for the rest of the arguments of `blockwiseModules()`. WGCNA identified 78 modules (Supplementary Figure S4 and Supplementary Table S1). WGCNA could not confidently assign 2,596 genes to any of the modules because they were hardly similar (associated) to any other gene. They were designated as Module 0.

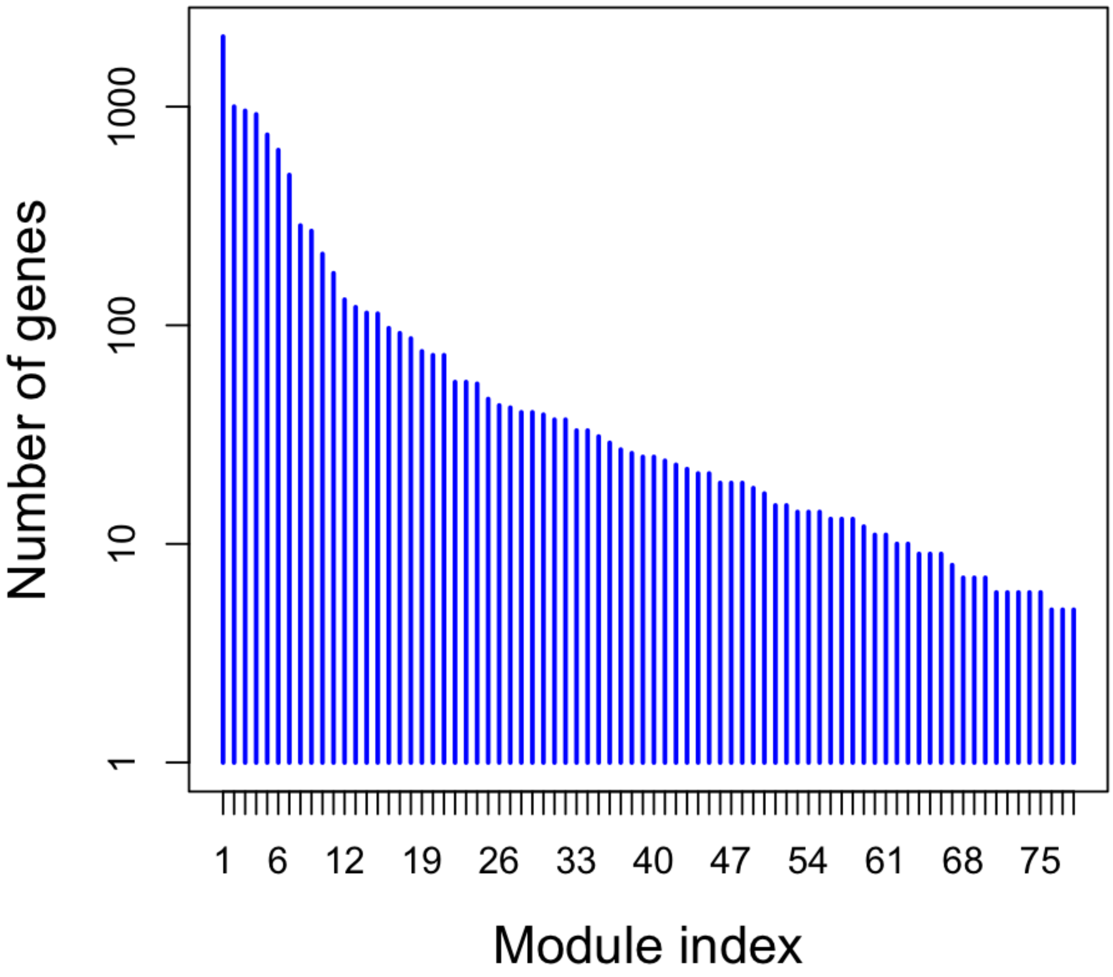

Supplementary Figure S4. The distribution of module sizes. The 78 modules are sorted on the x-axes based on their sizes. The largest and smallest modules consist of 2,092 and 5 genes, respectively. Module sizes have a mean, median, and standard deviation of 127, 25, and 310, respectively.

Supplementary Table S1: Module assignments. For each gene, the columns include: the gene symbol, the Entrez ID, the module to which the gene is assigned, and the weight of the gene in the module. In each module, genes are sorted based on the absolute value of their weights. A negative weight corresponds to anticorrelation between the eigengene of the module and the gene expression and DNA methylation levels.

Supplementary Note S5: Computing eigengenes. An eigengene of a module is a weighted average of the expression levels of all the genes in that module. These weights are adjusted so that the loss in the biological information is minimized[78,83]. To compute eigengenes, we used principal component analysis (PCA)[78]. Similar to our approach in Zainulabadeen et al[74], we used an oversampling procedure to equalize contributions of the high–risk and low–risk cases. There are 176 AML cases with both gene expression and DNA methylation data in the TCGA dataset. According to cytogenetic data, 32 cases are in the favorable–risk group and 40 cases are in the poor–risk groups. We balanced the number of favorable and poor cases using oversampling, so that both risk groups had comparable representatives in the analysis. That is, we repeated the data for each favorable and poor case 11 and 9 times, respectively. This resulted in 352 favorable samples and 360 poor samples. Then, we applied the `moduleEigengenes()` function from the WGCNA package to the oversampled gene expression data. This function computed the first principal component of each module, which maximized the explained variance, thus ensuring that loss of biological information was minimized[83]. Be reminded that the first principal component is a weighted average of gene expression levels[78]. We used the `project.eigen` function from the Pigengene package[84] (Version 1.4.2) to infer the eigengene values for the cytogenetically intermediate–risk group (Supplementary Table S2). This function computed a weighted average of gene expression levels using the same weights that were obtained from PCA. Some of the genes in modules 0, 1, 2, 4, 6, 10, and 11 had no variation in their expression levels. This led to undefined values for the corresponding inferred eigengenes. Therefore, we did not include these eigengenes in our analysis. We also used this approach to infer the eigengene values in the Beat AML dataset.

Inferring eigengenes in the AMLCG dataset This dataset was generated using three platforms. We combined the expression profiles of cases that were assayed using Affymetrix Human Genome U133A and U133B Arrays, and inferred the eigengene corresponding to each module using the `project.eigen` function from the Pigengene package[85]. The expression levels in the third platform (i.e., Affymetrix Human Genome U133 Plus 2.0 Array) were in a different scale compared to U133A and U133B Arrays, and thus could not be directly combined. To address this issue, we separately inferred eigengenes for samples that were assayed using the U133 Plus 2.0 platform, and then combined the resulting eigengenes from all three platforms.

Supplementary Table S2: Eigengenes. There is a table for each of the TCGA, AMLCG, and BEAT datasets. Rows correspond to an AML sample. Each of the ME0 to ME78 columns is an eigengene corresponding to one of the identified modules.

Supplementary Note S6: Details of survival analysis. We used an approach similar to our previous study on melanoma[74] to perform a Cox regression analysis, and also to fit a accelerated failure time (AFT) model to the 78 eigengenes data. From the 154 AML cases for

whom vital status, DNA methylation, and gene expression data were available in the TCGA dataset, 93 cases died of AML (mean = 1.1, median = 0.8, and standard deviation = 1 years), and 61 cases were alive at the last follow–up time (mean = 2.5, median = 2, and standard deviation = 2 years).

We used the `glmnet()` function from the glmnet package (Version 2.0-5)[43] to perform a penalized Cox regression analysis[86,87]. We set $\alpha = 1$ to use the least absolute shrinkage and selection operator (Lasso)[88]. The Lasso, also known as L_1 regularization, enforces most of the coefficients of the covariates (eigengenes) in the Cox proportional hazards model to be zero. Thereby, it identifies the modules that are the most associated with survival. Cox regression analysis selected Modules 46, 51, and 55.

We used the `survreg` function from the survival package (Version 2.39-4)[89] to fit an accelerated failure time (AFT) model to the three selected eigengenes[90]. We set the Weibull distribution with `scale=1` as the baseline hazard function, and used the default values for the rest of the parameters. Based on the fitted accelerated failure time model, we predicted the survival time of each sample using the following approach.

We chose two thresholds for the predicted values that maximized the precision of low– and high–risk predictions. The samples that had a predicted survival time between the two thresholds were considered intermediate–risk. We used the `survfit` function to obtain a Kaplan-Meier survival curve for each of the risk groups[91]. We used the `survdiff` function to test whether the survival curves that correspond to high–risk and low–risk groups differ significantly. This function computed the log-rank p–value of the corresponding Mantel-Haenszel test[92].

Supplementary Table S3: Risk assessment. The risk based on our gene network analysis is reported per each studied case. For the TCGA dataset, columns include: age at initial pathologic diagnosis, gender, vital status (where 1 indicates death), the number of days till death or the last follow–up time, cytogenetic abnormality, risk based on cytogenetics, risk based on approach of Gerstung et al , LSC17 score, risk based on LSC17, and risk based on our gene network approach. For the AMLCG dataset, risks based on LSC17 score are reported. For the BEAT dataset, risks based on ELN2017 are reported.

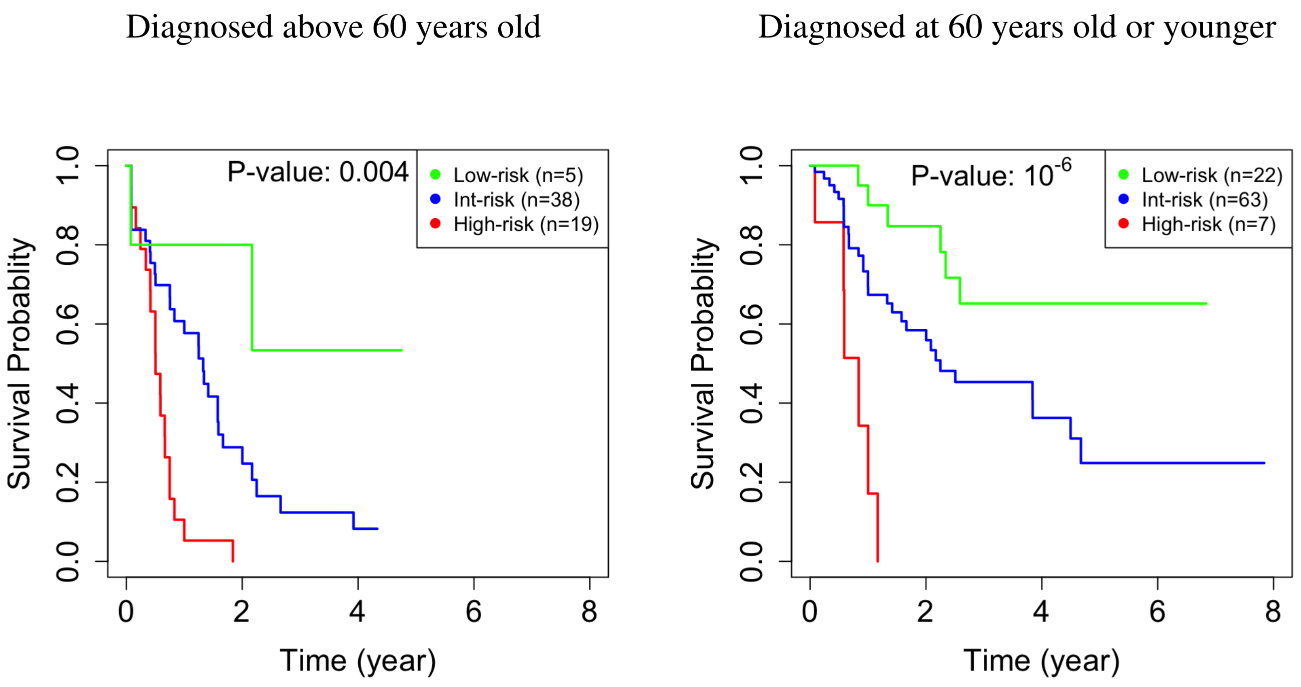

a **b**

Supplementary Figure S5. The KM curves for old and young groups. TCGA-AML cases were stratified into a group of 74 older patients, who were diagnosed over age of 60 (**a**), and another group of 92 relatively younger patients (**b**). The log–rank *p*–values indicate that differences between the low–risk group (green) and the high–risk group (red) are statistically in both age groups.

Supplementary Note S7: Comparison with LSC17 score. In the TCGA dataset, 59% of cases are categorized as intermediate–risk based on cytogenetic criteria, whereas, in the AMLCG 1999 dataset, 46% of cases are categorized as intermediate–risk based on ELN–2010. However, the survival rates of these cases varied (Figure 3a and Figure 4b). Therefore, prognostication of these "supposedly intermediate–risk" cases is an active area of AML research[6,38,47]. To show the usefulness of our approach in prognostication of cases that cannot be confidently prognosticated based on current clinical standards, we compared our risk assessment to the LSC17 score, which was recently developed by Ng et al[38] (Supplementary Note S1, Supplementary Figure S6 and Supplementary Table S3).

Specifically, for each case, we computed LSC17 score as the weighted average expression of the 17 genes. Cases with a score above the median were considered high–risk. The probability of 2–year survival for the subset with relatively higher risk based on LCS17 is above 0.2 in both datasets. The corresponding probability from our prediction is zero (Figure 3a and Figure 4b), which suggests our approach is more reliable than LSC17 in identifying high–risk cases. The 11 TCGA (Figure 3a) and 10 AMLCG 1999 (Figure 4b) cases that we determined to be high–risk are almost evenly distributed between the low– and high–risk groups based on the LSC17 score. This suggests the modules that we identified represent a signature that is distinct from the LSC17 score.

TCGA AMLCG

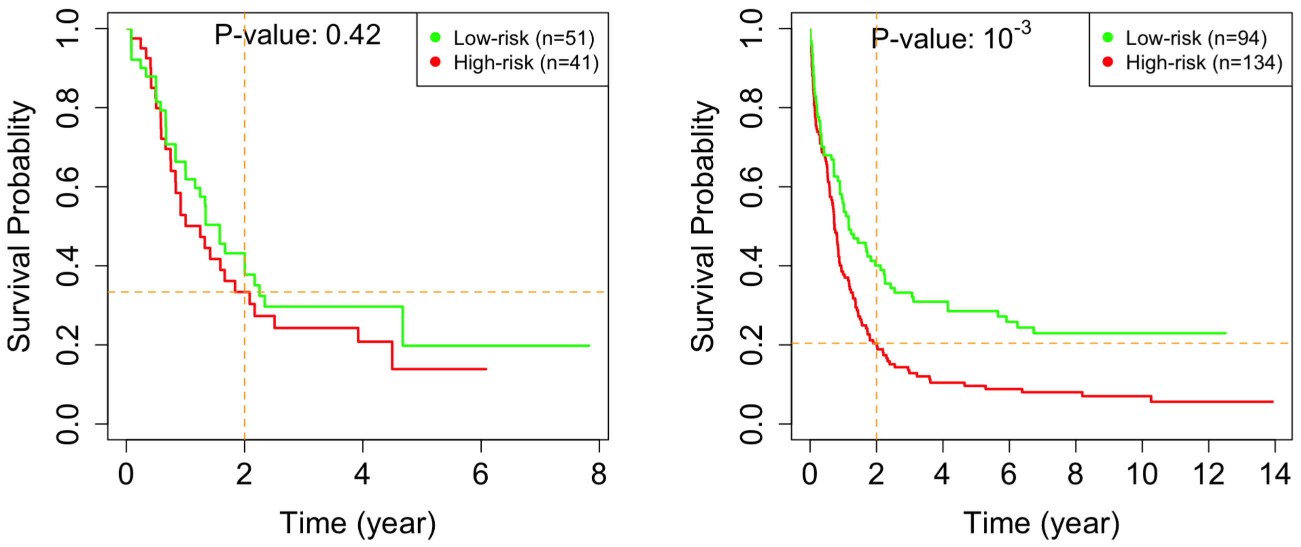

a **b**

Supplementary Figure S6. Performance of LSC17 on intermediate–risk cases. TCGA cases in the intermediate–risk category based on cytogenetic criteria were grouped into two subsets using their LSC17 scores, however, differences between the survival rates of these two subsets is not significant **(a)**. AMLCG cases with an intermediate ELN–2010 risk score of 2 or 3 were grouped into two subsets using their LSC17 score **(b)**. The probability of 2–year survival for the subset with relatively higher risk based on LCS17 score (red) is above 0.2 in both datasets. Nevertheless, the corresponding probability from our prediction is zero (Figure 3a and Figure 4b). Overall, the performance of LSC17 score seems to be limited in in its ability to identify truly high–risk cases among AML patients whose survival cannot be confidently predicted based on current standards in clinical practice.

References

1. Jemal A, Thomas A, Murray T, Thun M. Cancer statistics, 2002. CA: a cancer journal for clinicians. 2002;52(1):23–47.

2. Walter RB, Othus M, Paietta EM, Racevskis J, Fernandez HF, Lee JW, et al. Effect of genetic profiling on prediction of therapeutic resistance and survival in adult acute myeloid leukemia. Leukemia. 2015;29(10):2104.

3. Marcucci G, Haferlach T, Döhner H. Molecular genetics of adult acute myeloid leukemia: prognostic and therapeutic implications. Journal of Clinical Oncology. 2011;29(5):475–486.

4. Byrd JC, Mrózek K, Dodge RK, Carroll AJ, Edwards CG, Arthur DC, et al. Pretreatment cytogenetic abnormalities are predictive of induction success, cumulative incidence of relapse, and overall survival in adult patients with de novo acute myeloid leukemia: results from Cancer and Leukemia Group B (CALGB 8461): presented in part at the 43rd annual meeting of the American Society of Hematology, Orlando, FL, December 10, 2001, and published in abstract form. 59. Blood. 2002;100(13):4325–4336.

5. Grimwade D, Hills RK, Moorman AV, Walker H, Chatters S, Goldstone AH, et al. Refinement of cytogenetic classification in acute myeloid leukemia: determination of prognostic significance of rare recurring chromosomal abnormalities among 5876 younger adult patients treated in the United Kingdom Medical Research Council trials. Blood. 2010;116(3):354–365.

6. Papaemmanuil E, Gerstung M, Bullinger L, Gaidzik VI, Paschka P, Roberts ND, et al. Genomic classification and prognosis in acute myeloid leukemia. New England Journal of Medicine. 2016;374(23):2209–2221.

7. Patel JP, Gönen M, Figueroa ME, Fernandez H, Sun Z, Racevskis J, et al. Prognostic relevance of integrated genetic profiling in acute myeloid leukemia. New England Journal of Medicine. 2012;366(12):1079–1089.

8. Ley TJ, Ding L, Walter MJ, McLellan MD, Lamprecht T, Larson DE, et al. DNMT3A mutations in acute myeloid leukemia. New England Journal of Medicine. 2010;363(25):2424–2433.

9. Verhaak RG, Goudswaard CS, van Putten W, Bijl MA, Sanders MA, Hugens W, et al. Mutations in nucleophosmin (NPM1) in acute myeloid leukemia (AML): association with other gene abnormalities and previously established gene expression signatures and their favorable prognostic significance. Blood. 2005;106(12):3747–3754.

10. Döhner H, Estey EH, Amadori S, Appelbaum FR, Büchner T, Burnett AK, et al. Diagnosis and management of acute myeloid leukemia in adults: recommendations from an international expert panel, on behalf of the European LeukemiaNet. Blood. 2010;115(3):453–474.

11. Döhner H, Weisdorf DJ, Bloomfield CD. Acute myeloid leukemia. New England Journal of Medicine. 2015;373(12):1136–1152.

12. Arber DA, Orazi A, Hasserjian R, Thiele J, Borowitz MJ, Le Beau MM, et al. The 2016 revision to the World Health Organization classification of myeloid neoplasms and acute leukemia. Blood. 2016;127(20):2391–2405.

13. Valk PJ, Verhaak RG, Beijen MA, Erpelinck CA, van Doorn-Khosrovani SBvW, Boer JM, et al. Prognostically useful gene-expression profiles in acute myeloid leukemia. New England Journal of Medicine. 2004;350(16):1617–1628.

14. Bullinger L, Döhner K, Bair E, Fröhling S, Schlenk RF, Tibshirani R, et al. Use of gene-expression profiling to identify prognostic subclasses in adult acute myeloid leukemia. New England Journal of Medicine. 2004;350(16):1605–1616.

15. Verhaak RG, Wouters BJ, Erpelinck CA, Abbas S, Beverloo HB, Lugthart S, et al. Prediction of molecular subtypes in acute myeloid leukemia based on gene expression profiling. haematologica. 2009;94(1):131–134.

16. de Jonge HJ, Valk PJ, Veeger NJ, ter Elst A, den Boer ML, Cloos J, et al. High VEGFC expression is associated with unique gene expression profiles and predicts adverse prognosis in pediatric and adult acute myeloid leukemia. Blood. 2010;p. blood–2010.

17. Stegmaier K, Ross KN, Colavito SA, O'Malley S, Stockwell BR, Golub TR. Gene expression–based high-throughput screening (GE-HTS) and application to leukemia differentiation. Nature genetics. 2004;36(3):257.

18. Greiner J, Schmitt M, Li L, Giannopoulos K, Bosch K, Schmitt A, et al. Expression of tumor-associated antigens in acute myeloid leukemia: implications for specific immunotherapeutic approaches. Blood. 2006;108(13):4109–4117.

19. Tomasson MH, Xiang Z, Walgren R, Zhao Y, Kasai Y, Miner T, et al. Somatic mutations and germline sequence variants in the expressed tyrosine kinase genes of patients with de novo acute myeloid leukemia. Blood. 2008;111(9):4797–4808.

20. Taskesen E, Bullinger L, Corbacioglu A, Sanders MA, Erpelinck CA, Wouters BJ, et al. Prognostic impact, concurrent genetic mutations, and gene expression features of AML with CEBPA mutations in a cohort of 1182 cytogenetically normal AML patients: further evidence for CEBPA double mutant AML as a distinctive disease entity. Blood. 2011;117(8):2469–2475.

21. Metzelder S, Michel C, Von Bonin M, Rehberger M, Hessmann E, Inselmann S, et al. NFATc1 as a therapeutic target in FLT3-ITD-positive AML. Leukemia. 2015;29(7):1470.

22. Mills KI, Kohlmann A, Williams PM, Wieczorek L, Liu Wm, Li R, et al. Microarray-based classifiers and prognosis models identify subgroups with distinct clinical outcomes and high risk of AML transformation of myelodysplastic syndrome. Blood. 2009;114(5):1063–1072.

23. Haferlach T, Kohlmann A, Wieczorek L, Basso G, Te Kronnie G, Béné MC, et al. Clinical utility of microarray-based gene expression profiling in the diagnosis and subclassification of leukemia: report from the International Microarray Innovations in Leukemia Study Group. Journal of Clinical Oncology. 2010;28(15):2529–2537.

24. Gentles AJ, Plevritis SK, Majeti R, Alizadeh AA. Association of a leukemic stem cell gene expression signature with clinical outcomes in acute myeloid leukemia. Jama. 2010;304(24):2706–2715.

25. Klein HU, Ruckert C, Kohlmann A, Bullinger L, Thiede C, Haferlach T, et al. Quantitative comparison of microarray experiments with published leukemia related gene expression signatures. BMC bioinformatics. 2009;10:422.

26. Gaidzik VI, Bullinger L, Schlenk RF, Zimmermann AS, Röck J, Paschka P, et al. RUNX1 mutations in acute myeloid leukemia: results from a comprehensive genetic and clinical analysis from the AML study group. Journal of Clinical Oncology. 2011;29(10):1364–1372.

27. Kvinlaug BT, Chan WI, Bullinger L, Ramaswami M, Sears C, Foster D, et al. Common and overlapping oncogenic pathways contribute to the evolution of acute myeloid leukemias. Cancer research. 2011;p. canres–0176.

28. Bosman MCJ, Schepers H, Jaques J, Brouwers-Vos AZ, Quax WJ, Schuringa JJ, et al. The TAK1-NF-κB axis as therapeutic target for AML. Blood. 2014;p. blood–2014.

29. Eppert K, Takenaka K, Lechman ER, Waldron L, Nilsson B, Van Galen P, et al. Stem cell gene expression programs influence clinical outcome in human leukemia. Nature medicine. 2011;17(9):1086.

30. Li L, Li M, Sun C, Francisco L, Chakraborty S, Sabado M, et al. Altered hematopoietic cell gene expression precedes development of therapy-related myelodysplasia/acute myeloid leukemia and identifies patients at risk. Cancer cell. 2011;20(5):591–605.

31. De La Blétière DR, Blanchet O, Cornillet-Lefèbvre P, Coutolleau A, Baranger L, Geneviève F, et al. Routine use of microarray-based gene expression profiling to identify patients with low cytogenetic risk acute myeloid leukemia: accurate results can be obtained even with suboptimal samples. BMC medical genomics. 2012;5(1):6.

32. Li Z, Huang H, Li Y, Jiang X, Chen P, Arnovitz S, et al. Up-regulation of a HOXA-PBX3 homeobox-gene signature following down-regulation of miR-181 is associated with adverse prognosis in patients with cytogenetically-abnormal AML. Blood. 2012;p. blood–2011.

33. Li Z, Herold T, He C, Valk PJ, Chen P, Jurinovic V, et al. Identification of a 24-gene prognostic signature that improves the European LeukemiaNet risk classification of acute myeloid leukemia: an international collaborative study. Journal of Clinical Oncology. 2013;31(9):1172.

34. Xu J, Haigis KM, Firestone AJ, McNerney ME, Li Q, Davis E, et al. Dominant role of oncogene dosage and absence of tumor suppressor activity in Nras-driven hematopoietic transformation. Cancer discovery. 2013;p. CD–13.

35. Lavallée VP, Lemieux S, Boucher G, Gendron P, Boivin I, Armstrong RN, et al. RNA-sequencing analysis of core binding factor AML identifies recurrent ZBTB7A mutations and defines RUNX1-CBFA2T3 fusion signature. Blood. 2016;127(20):2498–2501.

36. Wilop S, Chou WC, Jost E, Crysandt M, Panse J, Chuang MK, et al. A three-gene expression-based risk score can refine the European LeukemiaNet AML classification. Journal of hematology & oncology. 2016;9(1):78.

37. Jung N, Dai B, Gentles AJ, Majeti R, Feinberg AP. An LSC epigenetic signature is largely mutation independent and implicates the HOXA cluster in AML pathogenesis. Nature communications. 2015;6:8489.

38. Ng SW, Mitchell A, Kennedy JA, Chen WC, McLeod J, Ibrahimova N, et al. A 17-gene stemness score for rapid determination of risk in acute leukaemia. Nature. 2016;540(7633):433.

39. Chuang MK, Chiu YC, Chou WC, Hou HA, Tseng MH, Kuo YY, et al. An mRNA expression signature for prognostication in de novo acute myeloid leukemia patients with normal karyotype. Oncotarget. 2015;6(36):39098.

40. Metzeler KH, Hummel M, Bloomfield CD, Spiekermann K, Braess J, Sauerland MC, et al. An 86-probe-set gene-expression signature predicts survival in cytogenetically normal acute myeloid leukemia. Blood. 2008;112(10):4193–4201.

41. Sarry JE, Murphy K, Perry R, Sanchez PV, Secreto A, Keefer C, et al. Human acute myelogenous leukemia stem cells are rare and heterogeneous when assayed in NOD/SCID/IL2Rγc-deficient mice. The Journal of clinical investigation. 2011;121(1):384–395.

42. Friedman J, Hastie T, Tibshirani R. Regularization paths for generalized linear models via coordinate descent. Journal of statistical software. 2010;33(1):1.

43. Simon N, Friedman J, Hastie T, Tibshirani R, et al. Regularization paths for Cox's proportional hazards model via coordinate descent. Journal of statistical software. 2011;39(5):1–13.

44. Ng SW, Mitchell A, Kennedy JA, Chen WC, McLeod J, Ibrahimova N, et al. Simultaneous identification and stratification of low molecular risk AML patients using a single LSC17-based Nanostring assay at diagnosis. Blood. 2017;540(Suppl 1):28–28.

45. Smith JL, Ries RE, Kolb A, Alonzo TA, Gerbing RB, Ma Y, et al. The LSC17 Leukemic Stem Cell Signature Predicts Outcome in Pediatric Acute Myeloid Leukemia. Blood. 2017;130(Suppl 1):24–24. Available from: http://www.bloodjournal.org/content/130/Suppl_1/24.

46. Tasian SK, Bornhäuser M, Rutella S. Targeting Leukemia Stem Cells in the Bone Marrow Niche. Biomedicines. 2018;6(1):22.

47. Gerstung M, Papaemmanuil E, Martincorena I, Bullinger L, Gaidzik VI, Paschka P, et al. Precision oncology for acute myeloid leukemia using a knowledge bank approach. Nature genetics. 2017;49(3):332–340.

48. Li E, Bestor TH, Jaenisch R. Targeted mutation of the DNA methyltransferase gene results in embryonic lethality. Cell. 1992;69(6):915–926.

49. Okano M, Bell DW, Haber DA, Li E. DNA methyltransferases Dnmt3a and Dnmt3b are essential for de novo methylation and mammalian development. Cell. 1999;99(3):247–257.

50. Jones PA, Taylor SM. Cellular differentiation, cytidine analogs and DNA methylation. Cell. 1980;20(1):85–93.

51. Glass JL, Hassane D, Wouters BJ, Kunimoto H, Avellino R, Garrett-Bakelman FE, et al. Epigenetic identity in AML depends on disruption of nonpromoter regulatory elements and

is affected by antagonistic effects of mutations in epigenetic modifiers. Cancer discovery. 2017;.

52. Figueroa ME, Lugthart S, Li Y, Erpelinck-Verschueren C, Deng X, Christos PJ, et al. DNA methylation signatures identify biologically distinct subtypes in acute myeloid leukemia. Cancer cell. 2010;17(1):13–27.

53. Lugthart S, Figueroa ME, Bindels E, Skrabanek L, Valk PJ, Li Y, et al. Aberrant DNA hypermethylation signature in acute myeloid leukemia directed by EVI1. Blood. 2011;117(1):234–241.

54. Deneberg S, Guardiola P, Lennartsson A, Qu Y, Gaidzik V, Blanchet O, et al. Prognostic DNA methylation patterns in cytogenetically normal acute myeloid leukemia are predefined by stem cell chromatin marks. Blood. 2011;p. blood–2011.

55. Negrotto S, Ng KP, Jankowska AM, Bodo J, Gopalan B, Guinta K, et al. CpG methylation patterns and decitabine treatment response in acute myeloid leukemia cells and normal hematopoietic precursors. Leukemia. 2012;26(2):244.

56. Rampal R, Alkalin A, Madzo J, Vasanthakumar A, Pronier E, Patel J, et al. DNA hydroxymethylation profiling reveals that WT1 mutations result in loss of TET2 function in acute myeloid leukemia. Cell reports. 2014;9(5):1841–1855.

57. Ferreira HJ, Heyn H, Vizoso M, Moutinho C, Vidal E, Gomez A, et al. DNMT3A mutations mediate the epigenetic reactivation of the leukemogenic factor MEIS1 in acute myeloid leukemia. Oncogene. 2016;35(23):3079.

58. Li S, Garrett-Bakelman FE, Chung SS, Sanders MA, Hricik T, Rapaport F, et al. Distinct evolution and dynamics of epigenetic and genetic heterogeneity in acute myeloid leukemia. Nature medicine. 2016;22(7):792.

59. Kelly AD, Kroeger H, Yamazaki J, Taby R, Neumann F, Yu S, et al. A CpG island methylator phenotype in acute myeloid leukemia independent of IDH mutations and associated with a favorable outcome. Leukemia. 2017;31(10):2011.

60. Kühnl A, Valk PJ, Sanders MA, Ivey A, Hills RK, Mills KI, et al. Down-regulation of the Wnt inhibitor CXXC5 predicts a better prognosis in acute myeloid leukemia. Blood. 2015;p. blood–2014.

61. Marcucci G, Yan P, Maharry K, Frankhouser D, Nicolet D, Metzeler KH, et al. Epigenetics meets genetics in acute myeloid leukemia: clinical impact of a novel seven-gene score. Journal of Clinical Oncology. 2014;32(6):548.

62. Qu Y, Lennartsson A, Gaidzik VI, Deneberg S, Karimi M, Bengtzén S, et al. Differential methylation in CN-AML preferentially targets non-CGI regions and is dictated by DNMT3A mutational status and associated with predominant hypomethylation of HOX genes. Epigenetics. 2014;9(8):1108–1119.

63. Luskin MR, Gimotty PA, Smith C, Loren AW, Figueroa ME, Harrison J, et al. A clinical measure of DNA methylation predicts outcome in de novo acute myeloid leukemia. JCI insight. 2016;1(9).

64. Bullinger L, Ehrich M, Döhner K, Schlenk RF, Döhner H, Nelson MR, et al. Quantitative DNA methylation predicts survival in adult acute myeloid leukemia. Blood. 2010;115(3):636–642.

65. Yalcin A, Kreutz C, Pfeifer D, Abdelkarim M, Klaus G, Timmer J, et al. MeDIP coupled with a promoter tiling array as a platform to investigate global DNA methylation patterns in AML cells. Leukemia research. 2013;37(1):102–111.

66. Schoofs T, Rohde C, Hebestreit K, Klein HU, Göllner S, Schulze I, et al. DNA methylation changes are a late event in acute promyelocytic leukemia and coincide with loss of transcription factor binding. Blood. 2013;121(1):178–187.

67. Akalin A, Garrett-Bakelman FE, Kormaksson M, Busuttil J, Zhang L, Khrebtukova I, et al. Base-pair resolution DNA methylation sequencing reveals profoundly divergent epigenetic landscapes in acute myeloid leukemia. PLoS genetics. 2012;8(6):e1002781.

68. Network CGAR, et al. Genomic and epigenomic landscapes of adult de novo acute myeloid leukemia. The New England journal of medicine. 2013;368(22):2059.

69. Taskesen E, Havermans M, van Lom K, Sanders MA, van Norden Y, Bindels E, et al. Two splice factor mutant leukemia subgroups uncovered at the boundaries of MDS and AML using combined gene expression and DNA-methylation profiling. Blood. 2014;p. blood–2013.

70. Taskesen E, Babaei S, Reinders MM, de Ridder J. Integration of gene expression and DNA-methylation profiles improves molecular subtype classification in acute myeloid leukemia. Bmc Bioinformatics. 2015;16(4):S5.

71. Mortazavi A, Williams BA, McCue K, Schaeffer L, Wold B. Mapping and quantifying mammalian transcriptomes by RNA-Seq. Nature Methods. 2008;5(7):621–628.

72. Zhang B, Gaiteri C, Bodea LG, Wang Z, McElwee J, Podtelezhnikov AA, et al. Integrated systems approach identifies genetic nodes and networks in late-onset Alzheimer's disease. Cell. 2013;153(3):707–720.

73. Tu Z, Zhang B, Zhu J. Network Integration of Genetically Regulated Gene Expression to Study Complex Diseases. Integrating Omics Data. 2015;p. 88.

74. Zainulabadeen A, Yao P, Zare H. Underexpression of Specific Interferon Genes Is Associated with Poor Prognosis of Melanoma. PloS one. 2017;12(1):e0170025.

75. Bibikova M, Barnes B, Tsan C, Ho V, Klotzle B, Le JM, et al. High density DNA methylation array with single CpG site resolution. Genomics. 2011;98(4):288–295.

76. Assenov Y, Müller F, Lutsik P, Walter J, Lengauer T, Bock C. Comprehensive analysis of DNA methylation data with RnBeads. Nature methods. 2014;11(11):1138.

77. Pearson K. Note on regression and inheritance in the case of two parents. Proceedings of the Royal Society of London. 1895;58:240–242.

78. Jolliffe I. Principal component analysis. Hoboken, NJ: Wiley Online Library; 2002.

79. Csardi G, Nepusz T. The igraph software package for complex network research. InterJournal, Complex Systems. 2006;1695(5):1–9.

80. R Core Team. R: A Language and Environment for Statistical Computing. Vienna, Austria; 2017. Available from: http://www.R-project.org/.

81. Langfelder P, Horvath S. WGCNA: an R package for weighted correlation network analysis. BMC bioinformatics. 2008;9(1):559.

82. Zhang B, Horvath S. A general framework for weighted gene co-expression network analysis. Statistical applications in genetics and molecular biology. 2005;4(1).

83. Oldham MC, Horvath S, Geschwind DH. Conservation and evolution of gene coexpression networks in human and chimpanzee brains. Proceedings of the National Academy of Sciences. 2006;103(47):17973–17978.

84. Foroushani A, Agrahari R, Docking R, Chang L, Duns G, Hudoba M, et al. Large-scale gene network analysis reveals the significance of extracellular matrix pathway and homeobox genes in acute myeloid leukemia: an introduction to the Pigengene package and its applications. BMC Medical Genomics. 2017;10(1):16.

85. Zare H, et al.. Pigengene: Computing and using eigengenes. Bioconductor; 2016. Available from: https://bioconductor.org/packages/devel/bioc/html/Pigengene.html.

86. Cox DR. Regression models and life-tables. In: Breakthroughs in statistics. Springer; 1992. p. 527–541.

87. Gui J, Li H. Penalized Cox regression analysis in the high-dimensional and low-sample size settings, with applications to microarray gene expression data. Bioinformatics. 2005;21(13):3001–3008.

88. Tibshirani R. Regression shrinkage and selection via the lasso. Journal of the Royal Statistical Society Series B (Methodological). 1996;p. 267–288.

89. Therneau TM, Grambsch PM. Modeling survival data: extending the Cox model. Springer Science & Business Media; 2000.

90. Kalbfleisch JD, Prentice RL. The statistical analysis of failure time data. vol. 360. John Wiley & Sons; 2011.

91. Kaplan EL, Meier P. Nonparametric estimation from incomplete observations. Journal of the American statistical association. 1958;53(282):457–481.

92. Mantel N, Haenszel W. Statistical aspects of the analysis of data from retrospective studies. J natl cancer inst. 1959;22(4):719–748.
